# More than a mechanical problem: Dysphagia and new-onset ineffective esophageal motility after LINX® placement—a case report

**DOI:** 10.1016/j.ijscr.2025.111646

**Published:** 2025-07-10

**Authors:** Patricia Ruiz-Cota, Agustina Altolaguirre, Andres Fontaine-Nicola, Ryan C. Broderick, Santiago Horgan

**Affiliations:** Division of Minimally Invasive Surgery, Department of Surgery, University of California San Diego, 9300 Campus Point Dr. La Jolla, San Diego, CA 92037, USA

**Keywords:** Magnetic sphincter augmentation, LINX, Ineffective esophageal motility, Postoperative dysphagia, Gastroesophageal reflux disease, Manometry

## Abstract

**Introduction:**

Magnetic sphincter augmentation (MSA) with the LINX® device is an effective surgical option for gastroesophageal reflux disease (GERD), offering benefits such as preservation of belching and reduced gas bloat syndrome compared to Nissen fundoplication. However, postoperative dysphagia is common complication, occurring in approximately 15–30 % of patients.

**Case presentation:**

We report the case of a 66-year-old female who underwent laparoscopic hiatal hernia repair with concurrent LINX® device placement for GERD. Three weeks postoperatively, she developed progressive dysphagia that was initially responsive to corticosteroids but recurred. Barium swallow demonstrated mild narrowing at the gastroesophageal junction. Despite three serial endoscopic dilations, her symptoms persisted. High-resolution manometry (HRM) revealed new-onset ineffective esophageal motility (IEM), in contrast to her preoperative study, which showed normal esophageal peristalsis. The LINX device was surgically removed due to refractory symptoms. Post-removal, the patient experienced significant symptomatic improvement. Repeat HRM one year later showed improved motility findings.

**Discussion:**

This case illustrates the potential for MSA to contribute to the development of esophageal dysmotility in patients with subtle or predisposing motility abnormalities. Early postoperative dysphagia should prompt repeat manometric evaluation and consideration of device removal in refractory cases.

**Conclusion:**

MSA may lead to de novo IEM. Thorough preoperative evaluation and timely reassessment of postoperative symptoms are key to optimizing patient outcomes.

## Highlights

• New-onset ineffective esophageal motility after LINX placement.
• Preoperative motility appeared normal with preserved peristaltic reserve.
• Dysphagia persisted despite multiple dilations and required device removal.
• Post-removal manometry showed improved but borderline motility.
• Preoperative manometry is essential for MSA selection, as impaired motility is a contraindication.

## 1 Introduction

Antireflux surgery is a well-established treatment for gastroesophageal reflux disease (GERD), particularly for patients with refractory symptoms or those who wish to avoid lifelong dependence on proton pump inhibitors (PPIs) [1]. Traditionally, fundoplication procedures, such as Nissen and Toupet fundoplication, have been the mainstay surgical options. However, wide variability in surgical technique and the potential for side effects—such as gas–bloat syndrome and dysphagia—have prompted the development of alternative approaches [1,2].

Magnetic sphincter augmentation (MSA) with the LINX® device has emerged as a promising alternative to fundoplication, offering effective reflux control while preserving the ability to belch and vomit [3]. The device consists of a ring of magnetic beads implanted around the lower esophageal sphincter (LES), providing biomechanical augmentation of the gastroesophageal junction (GEJ). The magnetic forces are designed to be strong enough to maintain closure at rest, thereby increasing GEJ pressure and reducing reflux, yet weak enough to allow for normal swallowing [2].

Despite the benefits of MSA, postoperative dysphagia is a frequently reported complication, affecting approximately 15–30 % of patients [[4], [5], [6]]. Early dysphagia is often attributed to the formation of a fibrotic capsule around the device, which can restrict bead mobility and impair its function. To mitigate this risk, patients are encouraged to “exercise” the device by maintaining a regular diet during the early postoperative period, as prolonged liquid intake may reduce bead actuation and contribute to capsule formation [4].

Although MSA preserves physiologic functions such as belching and reduces gas bloat compared to fundoplication, it has also been associated with a higher incidence of postoperative dysphagia. A recent meta-analysis found significantly increased rates of dysphagia and need for dilation following MSA compared to fundoplication [7].

While early dysphagia typically improves with time, a subset of patients develops persistent symptoms that may necessitate endoscopic dilation or device removal. Several studies have identified potential predictors of persistent dysphagia, including impaired preoperative esophageal motility, <80 % effective swallows on manometry, and absence of a large hiatal hernia. These findings emphasize the importance of preoperative motility assessment and appropriate patient selection prior to LINX placement [5,6].

The 2023 SAGES Safety and Effectiveness Analysis further highlights the importance of proper patient selection, noting that morbid obesity (BMI >35 kg/m^2^), Barrett's esophagus, Los Angeles grade C or D esophagitis, and large hiatal hernias (>3 cm) are relative contraindications for LINX implantation [8].

High-resolution manometry (HRM) has provided valuable insights into the impact of MSA on esophageal motility [3]. Although uncommon, some patients develop new-onset or worsening ineffective esophageal motility (IEM), which may contribute to persistent dysphagia. The development of dysmotility may be attributed to increased LES pressure, altered esophageal peristaltic reserve, or exacerbation of preexisting motility dysfunction [3]. However, the true incidence of new-onset IEM following LINX implantation remains unclear, as this complication is rarely reported in the literature.

We present the case of a patient who developed severe IEM following LINX placement for GERD, despite having normal preoperative motility. Her clinical course, diagnostic findings, and management strategy offer insights into a rare complication and reinforce the importance of thorough preoperative assessment and individualized postoperative care.

This case was managed at an academic tertiary care center with a specialized minimally invasive surgery program and esophageal motility center. This work has work has been reported in line with the SCARE criteria [9].

## 2 Case presentation

### 2.1 Initial presentation

A 66-year-old female underwent laparoscopic hiatal hernia repair with magnetic sphincter augmentation (MSA) using a 15-bead LINX device for GERD. Three weeks later, she developed progressive dysphagia, which initially improved with corticosteroids but soon recurred. She reported difficulty swallowing solids, frequent regurgitation, and a sensation of food sticking in the chest.

Her medical history included esophageal spasms, based on a manometry study performed seven years earlier (unavailable for review), and a known Schatzki ring with multiple prior endoscopic dilations, the most recent approximately one year before LINX placement. Her surgical history included appendectomy, cesarean section, hysterectomy, and tubal ligation.

### 2.2 Preoperative evaluation

**Fig. 1:**
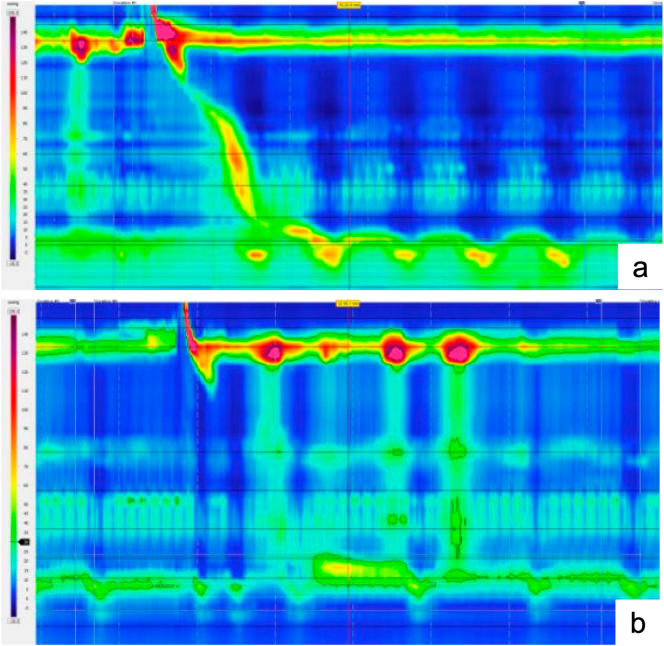
High-resolution manometry (HRM) before and after LINX® placement. (a) Preoperative HRM demonstrating normal LES relaxation (68 %), 80 % intact peristalsis, and preserved peristaltic reserve—consistent with normal esophageal motility. (b) Postoperative HRM at four months showing normal LES relaxation (68 %), with 60 % failed swallows and 100 % incomplete bolus clearance—findings consistent with IEM per the Chicago Classification v4.0.

Preoperative studies included a barium swallow, which demonstrated no mucosal or mural abnormalities but revealed delayed esophageal clearance with positional reflux and a small hiatal hernia, without tertiary contractions. There was no radiographic evidence of Schatzki's ring. High-resolution manometry (HRM) demonstrated normal LES relaxation (68 %), 80 % intact peristalsis, 20 % weak swallows, and preserved peristaltic reserve—findings consistent with normal motility (Fig. 1a). A 96-h Bravo pH study showed a total distal acid exposure time of 10.8 % and a DeMeester score of 36.8, with strong symptom correlation. As part of the Bravo capsule placement, an upper endoscopy was performed and showed no structural abnormalities in the esophagus aside from Los Angeles grade A esophagitis. Notably, no Schatzki's ring or residual scarring from prior dilations was identified. Her BMI was 28.3 kg/m^2^ at the initial consultation and 26.6 kg/m^2^ on the day of LINX placement.

### 2.3 Postoperative evaluation and therapeutic interventions

**Fig. 2:**
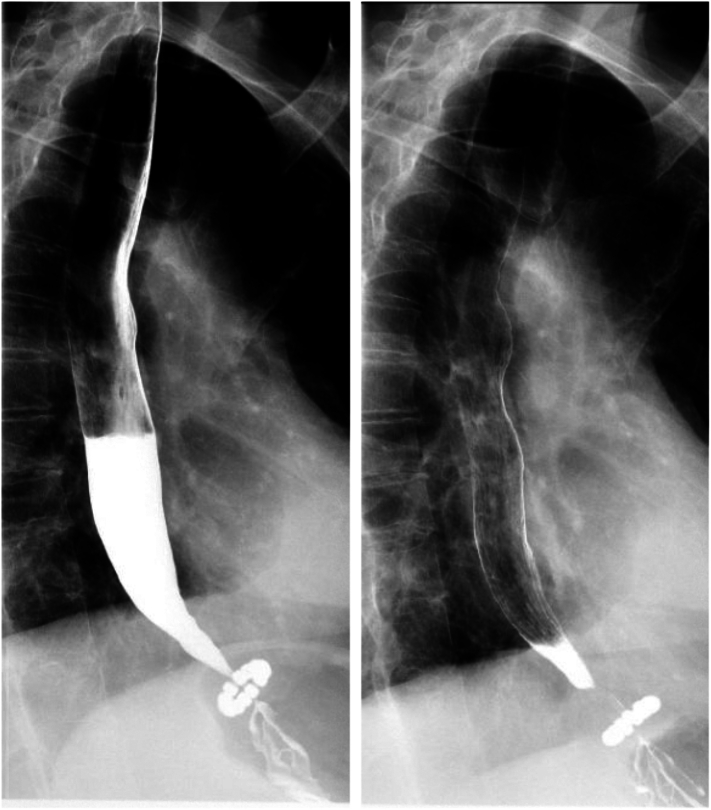
Postoperative barium swallow study three weeks after LINX® placement. LINX® device in expected position, with mild narrowing at the GEJ, mildly delayed contrast transit, and no tablet retention.

Due to dysphagia at three weeks after surgery, a postoperative barium swallow showed the LINX in position, with mild narrowing at the GEJ, mildly delayed contrast transit, and no tablet retention (Fig. 2). Mild tertiary contractions were also noted.

Based on these findings and the patient's significant symptomatology, an upper endoscopy with dilation to 54 French was performed at eight weeks postoperatively, resulting in transient relief lasting only one day. Due to recurrence of dysphagia, a second dilation to 54 French was performed two weeks later, again with minimal improvement. A third dilation to 56 French was performed one month later, but the patient continued to experience significant dysphagia despite these interventions.

HRM performed four months after LINX placement (Fig. 1b) showed normal LES relaxation (68 %), with 60 % failed swallows and 100 % incomplete bolus clearance. These findings were consistent with ineffective esophageal motility (IEM) per Chicago Classification v4.0, in contrast to the patient's normal preoperative motility.

Given these findings, LINX device removal was recommended. Intraoperatively, the device appeared intact and was removed without notable challenge or abnormal findings. The LINX was successfully divided and removed without complications. Postoperative recovery was uneventful, and the patient was discharged the following day on a soft diet and PPIs.

### 2.4 Follow-up and outcomes

**Fig. 3:**
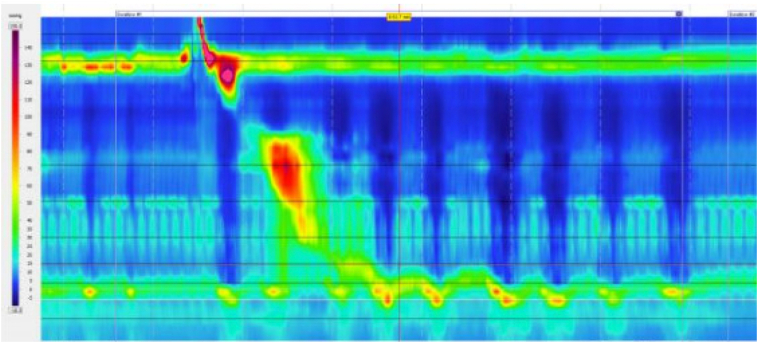
Follow-up HRM 12 months after LINX® removal. HRM shows 60 % intact peristalsis—borderline findings that do not meet the diagnostic criteria for IEM.

At one month, the patient reported symptomatic improvement. By two months, dysphagia had largely resolved, and she discontinued PPIs without recurrent reflux. Twelve months after removal, HRM demonstrated 60 % intact peristalsis—borderline findings that did not meet the diagnostic criteria IEM (Fig. 3).

A timeline summarizing the clinical course is presented in Table 1.

**Table 1:** Timeline summarizing the patient's preoperative evaluation, interventions, and outcomes over a 12-month period.

Timepoint	Event
T − 1 to 0 weeks	Preoperative evaluation: – Barium swallow: delayed clearance, hiatal hernia – HRM: 80 % intact peristalsis, normal motility. – Bravo pH: DeMeester score 36.8 (GERD diagnosis) – Upper endoscopy: no structural esophageal abnormalities
T0	Laparoscopic hiatal hernia repair and LINX placement
+3 weeks	Onset of progressive dysphagia (initially improved with corticosteroids)
+8 weeks	First endoscopic dilation (54 Fr, transient relief)
+10 weeks	Second dilation (54 Fr, minimal improvement)
+14 weeks	Third dilation (56 Fr, no significant improvement)
+4 months	HRM: new-onset IEM (60 % failed swallows, 100 % incomplete clearance)
+5 months	LINX removal (intact device, no erosion or inflammation)
+6 months	Symptomatic improvement; off PPIs
+12 months	Follow-up HRM: improved motility (60 % intact, no longer met IEM diagnostic criteria)

GERD; gastroesophageal reflux disease; HRM, high-resolution manometry; IEM, ineffective esophageal motility.

## 3 Discussion

Dysphagia is the most frequently reported complication following MSA, particularly in the early postoperative period. While often transient, persistent dysphagia can significantly impact patient quality of life and may necessitate endoscopic dilation or device removal. Identifying patients at risk and understanding the underlying mechanisms of dysphagia remain important challenges in patient selection and postoperative care.

Ayazi et al. identified three independent predictors of persistent postoperative dysphagia after MSA: absence of a large hiatal hernia, presence of preoperative dysphagia, and <80 % peristaltic contractions on preoperative HRM [5]. They proposed that patients without hiatal hernias may perceive greater resistance after MSA due to the absence of pre-existing obstruction, whereas hernia repair may alleviate baseline outflow resistance in other patients. Additionally, impaired preoperative esophageal motility or visceral hypersensitivity may predispose patients to symptom persistence after device implantation. In the same study, having <80 % effective swallows was strongly predictive of postoperative dysphagia, emphasizing the importance of evaluating esophageal functional reserve prior to MSA.

Bologheanu et al. further demonstrated that device size may influence outcomes. In their cohort, placement of a 13-bead LINX device was the only independent predictor of persistent dysphagia [6]. This supports the notion that device sizing should be approached cautiously, as excessive tension may exacerbate postoperative symptoms. Although our patient received a 15-bead device, her symptoms were refractory to three dilations, indicating that factors beyond size—such as altered motility or esophageal hypersensitivity—may be more relevant in select patients.

Comparative studies have also shown that MSA is associated with higher rates of postoperative dysphagia than laparoscopic Nissen fundoplication. In a recent systematic review and meta-analysis by Fadel et al., MSA was significantly associated with increased dysphagia (WMD 0.41; 95 % CI: 0.17–0.65; *P* = 0.001) and a higher need for postoperative dilations (WMD 0.11; 95 % CI: 0.02–0.21; *P* = 0.013) compared to fundoplication [7]. However, MSA demonstrated superior functional outcomes, including preservation of belching and emesis, and a lower incidence of gas bloat—factors that may enhance patient satisfaction despite the elevated risk of dysphagia.

Our patient had a small hiatal hernia that was repaired at the time of MSA implantation. The decision to proceed with MSA rather than fundoplication was based on shared decision-making. The patient preferred a less invasive option that preserved belching and vomiting and wished to avoid potential side effects of fundoplication, such as gas-bloat syndrome. Preoperative manometry showed normal LES relaxation pressure and 80 % intact peristalsis. Postoperatively, she developed new-onset IEM, with persistent dysphagia unresponsive to serial dilations. She ultimately required LINX explantation, after which she experienced significant symptomatic improvement. A follow-up manometry performed one year later showed improved, with no longer diagnostic IEM criteria. This case illustrates the complex interplay between preoperative motility, patient perception, mechanical resistance imposed by the device, and scar tissue development around the device. This highlights the importance of careful preoperative assessment and individualized postoperative management.

Despite the increasing use of MSA and recognition of dysphagia as a common postoperative complication, there remains a lack of standardized guidelines for its management. While Ayazi et al. have proposed a stepwise algorithm incorporating symptom duration, preoperative motility, and endoscopic findings [5], there is insufficient evidence to support its widespread adoption. A recent MAUDE database analysis by McMillan et al. highlighted the broad spectrum of adverse outcomes following LINX implantation, including persistent dysphagia, erosion, migration, and device malfunction [10]. In retrospect, the patient's history of Schatzki ring and repeated dilations (even with no evidence in preoperative studies) may have reflected subtle underlying motility dysfunction or contributed to impaired bolus transit, despite relatively preserved preoperative peristalsis. We also suspect that a fibrotic reaction around the device may have further reduced esophageal compliance, limiting the effectiveness of serial dilations. Further prospective studies are needed to establish validated protocols for the assessment and management of post-LINX dysphagia. This case reinforces the key role of esophageal motility reserve in patient selection for MSA and indicates that early postoperative dysphagia should prompt timely reassessment.

## 4 Conclusion

This case highlights the potential for MSA to contribute to the development of esophageal motility abnormalities, resulting in persistent dysphagia. Careful preoperative manometric assessment and postoperative symptom monitoring are essential. In refractory cases, early intervention—including device removal—may be warranted to prevent prolonged morbidity and improve outcomes.

## CRediT authorship contribution statement

PRC contributed to concept, data collection, drafting, and final manuscript editing. AA, AFN, SH, and RCB contributed to case management, critical revision, and final approval. All authors read and approved the final manuscript.

## Consent

Written informed consent was obtained from the patient for publication and any accompanying images. A copy of the written consent is available for review by the Editor-in-Chief of this journal on request.

## Ethical approval

Ethical approval was not required for this case report, as case reports involving a single patient are deemed not to constitute human subjects research according to institutional policies.

## Guarantor

Patricia Ruiz Cota, MD.

## Registration of research studies

1.Name of the registry: NA.

2.Unique identifying number or registration ID: NA.

3.Hyperlink to your specific registration (must be publicly accessible and will be checked):

## Sources of funding

We did not receive any financial support for this study.

## Declaration of competing interest

The authors declare that they have no conflicts of interest related to this case report.
